# The usual Interstitial pneumonia pattern in autoimmune rheumatic diseases

**DOI:** 10.1186/s12890-023-02783-z

**Published:** 2023-12-11

**Authors:** Fabrizio Luppi, Andreina Manfredi, Paola Faverio, Michael Brun Andersen, Francesca Bono, Fabio Pagni, Carlo Salvarani, Elisabeth Bendstrup, Marco Sebastiani

**Affiliations:** 1 Respiratory Disease, Fondazione IRCCS San Gerardo dei Tintori, Monza, Italy; 2 School of Medicine and Surgery, University of Milano-Bicocca, Milan, Italy; 3 Rheumatology Unit, University of Modena and Reggio Emilia, Azienda Ospedaliero-Universitaria Policlinico di Modena, Modena, Italy; 4 Copenhagen University Hospital Gentofte, Copenhagen, Denmark; 5 Department of Clinical Medicine, Copenhagen University, Copenhagen, Denmark; 6 Pathology, Fondazione IRCCS San Gerardo dei Tintori, Monza, Italy; 7 Rheumatology Unit, Dipartimento Medicina Interna e Specialità Mediche, Azienda Unità Sanitaria Locale di Reggio Emilia-Istituto di Ricerca e Cura a Carattere Scientifico, Reggio Emilia, Italy; 8 Center for Rare Lung Disease, Department of Respiratory Diseases and Allergy, Aarhus University Hospital, and Department of Clinical Medicine, Aarhus University, Aarhus, Denmark; 9 Department of Clinical Medicine, Aarhus University, Aarhus, Denmark

**Keywords:** Interstitial lung disease, Usual interstitial pneumonia, Autoimmune rheumatic disease, Rheumatoid arthritis, Diagnosis, Immunomodulatory drug, Antifibrotic treatment

## Abstract

Usual Interstitial Pneumonia (UIP) is characterized by progression of lung parenchyma that may be observed in various autoimmune rheumatic diseases (ARDs), including rheumatoid arthritis and connective tissue diseases. From a diagnostic point of view, a UIP pattern related to ARDs may display imaging and pathological features able to distinguish it from that related to IPF, such as the “straight-edge” sign at HRCT and lymphoplasmacytic infiltrates at histologic specimens. Multidisciplinary approach (MDD), involving at least pulmonologist, rheumatologist and radiologist, is fundamental in the differential diagnosis process, but MDD is also required in the evaluation of severity, progression and response to treatment, that is based on the combination of changes in symptoms, pulmonary function trends, and, in selected patients, serial CT evaluation. Differently from IPF, in patients with ARDs both functional evaluation and patient-reported outcomes may be affected by systemic involvement and comorbidities, including musculoskeletal manifestations of disease. Finally, in regards to pharmacological treatment, immunosuppressants have been considered the cornerstone of therapy, despite the lack of solid evidence in most cases; recently, antifibrotic drugs were also proposed for the treatment of progressive fibrosing ILDs other than IPF. In ARD-ILD, the therapeutic choice should balance the need for the control of systemic and lung involvements with the risk of adverse events from multi-morbidities and -therapies. Purpose of this review is to summarize the definition, the radiological and morphological features of the UIP pattern in ARDs, together with risk factors, diagnostic criteria, prognostic evaluation, monitoring and management approaches of the UIP-ARDs.

## Introduction

Interstitial lung diseases (ILDs) encompass a heterogenous group of pulmonary parenchymal disorders that are classified together because of similarities in their clinical presentation, chest radiographic appearance and physiologic features and may ultimately lead to pulmonary fibrosis and early death [1].

ILDs contain several categories with different prognoses including those characterized by environmental exposure, such as asbestosis, hypersensitivity pneumonitis or drug-induced ILDs, granulomatous disorders like sarcoidosis, idiopathic interstitial pneumonias (IIPs), as idiopathic pulmonary fibrosis (IPF) and autoimmune rheumatic diseases (ARD)-associated interstitial lung disease (ARD-ILD) [2]. Patients with ARD-associated ILD constitutes approximately 20% of all patients with ILD [3]. In general, ILD results from inflammation and/or excessive accumulation of connective tissue matrix in the lung interstitium, but all tissues within the lung can be affected [4]; the disease is usually triggered by specific and generally environmetal and genetic risk factors, activating distinct pathways that drive fibrosis of differing histological patterns in individuals who are genetically susceptible.

Currently, the only available classification of ARD-ILD is based on the histological classification of IIPs. All histological patterns seen in IIPs are also reported to occur in ARD-ILD [2]. However, the relative prevalence and prognostic relevance of histological patterns differ greatly between idiopathic and ARD-ILDs [5]. In idiopathic diseases, usual interstitial pneumonia (UIP), clinically corresponding to IPF, is the most prevalent pattern [6]. IPF has a worse prognosis than other ILDs, including fibrotic nonspecific interstitial pneumonia (NSIP), the other predominantly fibrotic idiopathic disease in ARD-ILD [7].

The UIP is a pathologic diagnostic term introduced in 1969 by Liebow and Carrington as part of the early classification of interstitial pneumonias [8]. In this initial classification, if a biopsy did not present features of desquamative interstitial pneumonia, bronchiolitis obliterans interstitial pneumonia, lymphoid interstitial pneumonia, or giant cell interstitial pneumonia, it was categorized into the category of UIP [8]. In 1969, the natural course of UIP was described with epithelial necrosis, progressing through diffuse alveolar damage, and then either resolving or progressing to interstitial proliferation and eventually honeycomb “end-stage” lung fibrosis [9]. About twenty years ago, UIP was clarified to be a chronic fibrosing interstitial lung disease that was clinically associated to IPF, and was defined as the clinical diagnostic term only to be used in the setting of patients with chronic fibrosing lung disease and a surgical biopsy showing UIP [10]. The vast array of clinical terms used for the idiopathic progressive fibrotic lung disease that showed histologic features of UIP underscores the challenges with nomenclature around the turn of the millennium [11].

In 2011, the clinical practice guidelines for the diagnosis of IPF from the American Thoracic Society/European Respiratory Society/Japanese Respiratory Society/Latin American Thoracic Association fundamentally changed the work-up of patients with suspected IPF and also the structure of the histologic criteria for the pathologic diagnosis of UIP [12]. The 2011 guidelines were the first to introduce the concept of a multidisciplinary diagnosis of IPF in patients *without* a surgical lung biopsy (SLB) if the patient had the characteristic clinical presentation, and a high-resolution computed tomography (HRCT) scan showing radiologic UIP according to the guidelines. In addition, the guidelines recognize the importance of linking the histologic diagnosis of UIP with clinical IPF and have thus provided the ability to assign a probability score for UIP based on histologic features (UIP, probable UIP, possible UIP, and not UIP in 2011, revised to UIP, probable UIP, indeterminate for UIP, and alternative diagnosis in 2018) [13].

Although with a different prevalence, various ILDs, including ARD-ILD, show a UIP pattern at high-resolution computed tomography (HRCT) and/or histology, indicating that UIP is rather a stereotypic response of lung tissue to different chronic injuries. Indeed, the UIP pattern may be observed in various ARDs, including rheumatoid arthritis (RA), systemic sclerosis (SSc) and anti-synthetase disorders, but also in other ILDs, including fibrotic hypersensitivity pneumonia and asbestos exposure, sarcoidosis, familiar forms of pulmonary fibrosis (Hermansky–Pudlak syndrome, and genetic diseases involving surfactant proteins or telomerase complex (TERT, TERC, RTEL1, PARN, or DKC1 mutations) and drug toxicity [2]. Irrespective of its association, UIP generally shows the worst survival outcomes, whether occurring in idiopathic or non-idiopathic interstitial pneumonias, including ARD-ILDs [6]. Further supporting this, recent studies demonstrated that the gain-of-function MUC5B promoter variant rs35705950 initially observed in IPF [14], was also associated with fibrotic hypersensitivity pneumonia and RA-related UIP and to a worse outcome [15], while this was not the case in other ARDs [16–18].

Instead, a NSIP pattern is the most prevalent pattern in ILD associated with SSc [19], polymyositis, dermatomyositis [20] and primary Sjögren’s syndrome (pSS) [21].

Although ILD is considered a major cause of morbidity and mortality of patients with ARDs [22], the clinical course of ARD-ILD is variable with a proportion of ARD-ILD patients developing a progressive pulmonary fibrosis (PPF) phenotype characterized by a rapid deterioration of pulmonary function tests (PFTs), leading to respiratory failure and higher mortality [23–25]. The presence of an UIP pattern per se seems to be a risk factor for a progressive phenotype in ARD-UIP [18].

In this review, we aim to summarize the definition and the radiological and morphological features of the UIP pattern in ARDs, risk factors, diagnostic criteria, prognostic evaluation, monitoring approach and management.

## Pathology of the UIP pattern

**Fig. 1:**
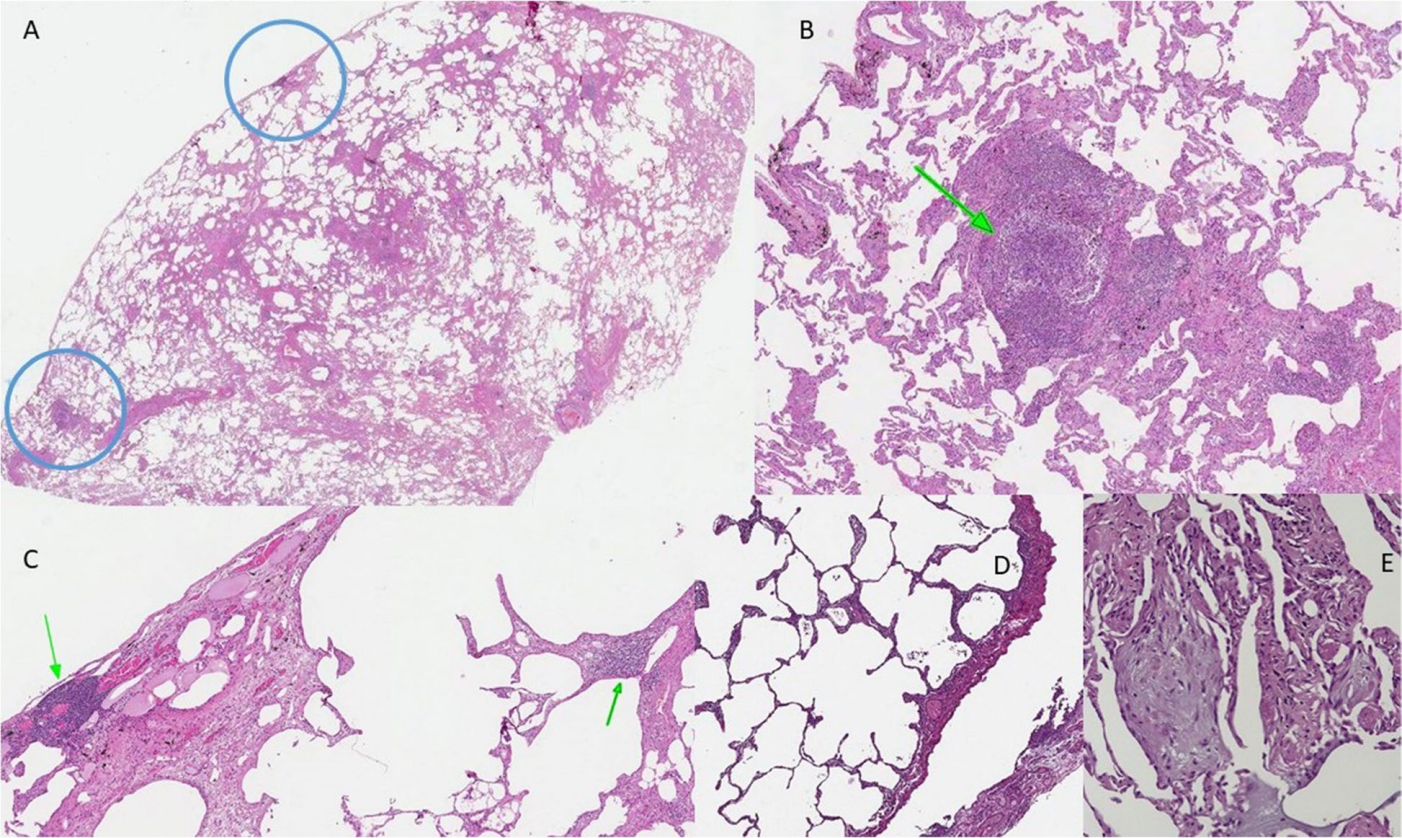
Pathological ancillary findings in ARDs-related UIP pattern **A**] Overview of a surgical biopsy: normal lung architecture is distorted by fibrosis with UIP pattern (H&E, 20x). **B**] The same biopsy, at higher magnification: evidence of nodular hyperplastic germinal centre, interstitial and subpleural located **C**] (green arrows, H&E, 150x; 100x). **D**] Slight septal chronic infiltrate, in association with pleuritis in an almost normal lung parenchyma spared by fibrosis (H&E, 100x). **E**] Fibroblastic polyps with endoalveolar projection in a OP pattern (H&E, 200x)

Although the histology in ARD-ILD is heterogeneous, a UIP pattern is also found in ARDs, more frequently in RA and ANCA-associated vasculitis (Fig. 1A). Moreover, it can also be observed in other diseases such as SSc [26], Sjogren’s syndrome [27] and more rarely antisynthetase syndrome [20].

A UIP histologic pattern is defined as a fibrotic and irreversible remodeling of the lung parenchyma. The aim of both 2011 and 2018 IPF guidelines was linking the histologic diagnosis of UIP with the clinical syndrome of IPF, finally assigning a probability score for UIP [13].

The hallmark features characterizing the UIP pattern include dense fibrosis with architectural distortion (i.e., destructive scarring and/or honeycombing), fibrosis localized in peripheral areas of lung lobular architectures and spatial and temporal heterogeneity with presence of fibroblast foci and absence of features to suggest an alternate diagnosis. The distribution of fibrosis is particularly relevant in UIP: it starts from the periphery of the lobules and surrounds the centrilobular regions, leaving unscathed areas of parenchyma in the typical patchy way.

Fibroblastic foci are defined as subepithelial and interstitial discrete areas, in which fibroblasts are organized in a linear way within a pale staining matrix. They are often located at the border of scarred areas. Honeycombing is defined as cystic spaces filled with mucous and inflammatory cells debris, covered by respiratory epithelium and embedded in advanced fibrosis; in comparison to the radiologically required signs, honeycombing is not mandatory for the diagnosis of a definite histological UIP pattern, and furthermore, when extensive and replacing the whole parenchyma, is a histologic hallmark of end-stage lung disease from any cause (not only IPF) and it is therefore classified as “probable UIP”.

ARDs, particularly in the case of RA, can show in a pattern of pulmonary fibrosis very similar to that observed in typical UIP in IPF [28]; particularly, the presence of the patchy distribution of the fibrosis and the presence of fibroblast foci is characteristic.

Some ancillary findings can help to distinguish UIP-ARD from idiopathic UIP: the first is the presence of dense lymphoplasmacytic infiltrates, and particularly, a nodular lymphoid hyperplasia with prominent germinal centre (Fig. 1B).

Honeycombing can be associated to chronic lymphocytic inflammation as well, but in case of lymphoid aggregates, and especially in association with prominent hyperplastic germinal centre, the suspicion of an ARD increases. Another feature of ARD-ILD is pleural involvement with chronic, sometimes fibrosing pleuritis (Fig. 1C, D).

The non-fibrotic lung parenchyma may also show a slight lymphocyte infiltrate, a subtle but suggestive sign of an ARD background in association to the previously described, resulting in an overlapping pattern of NSIP and UIP. Another typical feature of ARD-ILD is the association of different patterns within the same specimen (Fig. 1D), including the overlap with organizing pneumonia (OP). In this case, some OP polyps with typical endoalveolar projection may be associated with the UIP and/or NSIP pattern (Fig. 1E). In table 1 are summarized the radiological and pathological ancillary findings observed in ARD-UIP (Table 1).

**Table 1:** Radiological and pathological signs suggestive of an interstitial lung disease in the framework of an autoimmune disease

	Ancillary finding	Definition
Radiological	Anterior upper lobe sign	Concomitant fibrosis with honeycombing in the anterior part of the upper lobe combined with a UIP pattern with a predominant basal distribution
	Straight-edge sign	Sparing of the upper and midzones from fibrosis with a horizontal demarcation between fibrotic and normal lung parenchyma, without cranial extension of fibrosis along the lateral chest wall
	Exuberant honeycombing	Honeycomb cysts comprising more than 70% of the fibrotic lung areas.
Pathological	Dense lymphoplasmacytic infiltrates	Nodular aggregates of limphocytes often with a prominent germinal centre
	Pleuritis	Unexplained pleural inflammation of the serosal surfaces (with or without hyperplastic germinal centre)
	Nonspecific interstitial pneumonia	Non-fibrotic lung parenchyma may show a slight lymphocyte infiltrate
	Organizing pneumonia	Organizing pneumonia polyps with typical endoalveolar projection
	Overlap patterns (UIP/NSIP/OP)	Association of different patterns variously combined

A pathological diagnosis of a UIP pattern entails the need to discriminate between a primary or secondary UIP, searching for ancillary findings and assigning a specific diagnosis (for example, granulomas in a UIP pattern suggest a hypersensitivity pneumonia, as well as marked lymphoid follicular hyperplasia and/or pleurisy may suggest an ARD). However, in most of the cases, an ARD-UIP pattern may be identical to that observed in IPF, without pathognomonic findings suggesting alternative etiologies [29]. Subsequently, a UIP histologic pattern requires a multidisciplinary discussion aiming to confirm the diagnosis, and defining an appropriate management.

## Radiology of the UIP pattern

The radiological UIP pattern has been defined by both the Fleischner Society [30] and within the official ATS/ERS/JRS/ALAT statement [13] and include reticulation, traction bronchiectasis/bronchiolectasis and honeycombing as the hallmark features characterizing the pattern combined with a predominant subpleural distribution with a cranio-caudal gradient. However, a more diffuse or even asymmetrical distribution does not exclude the pattern of UIP. The probable UIP pattern is characterized by the absence of honeycombing, while the indeterminate UIP pattern is defined as fibrosis not meeting criteria for a UIP or probable UIP pattern and without features suggesting an alternative diagnosis. These include upper or mid zone predominant fibrosis, marked mosaic attenuation, air trapping, ground glass opacification with subpleural sparing, widespread pleural plaques, multiple pulmonary nodules or markedly enlarged lymph nodes [13].

*Reticulation* is defined as the irregular/coarse thickening of inter- and intralobular septa while *ground glass* may be seen in combination with features of fibrosis and it is interpreted as fine reticulation beyond the spatial resolution of modern CT systems. *Traction bronchiectasis/bronchiolectasis* constitutes a spectrum from subtle irregularity of the wall to distortion of the airway and is regarded as a precursor to formation of *honeycomb cysts*. *Honeycombing* is clustered cystic airspaces usually ranging from 3 to 10 mm, but occasionally as large as 25 mm representing peripheral dilatation of airways with thickening of the walls.

A UIP pattern related to ARDs in some cases displays imaging features that distinguish it from a UIP pattern related to IPF; specifically, the “straight-edge” sign, the “exuberant honeycombing” sign and the “anterior upper lobe” sign are suggestive for an autoimmune background [31].

**Fig. 2:**
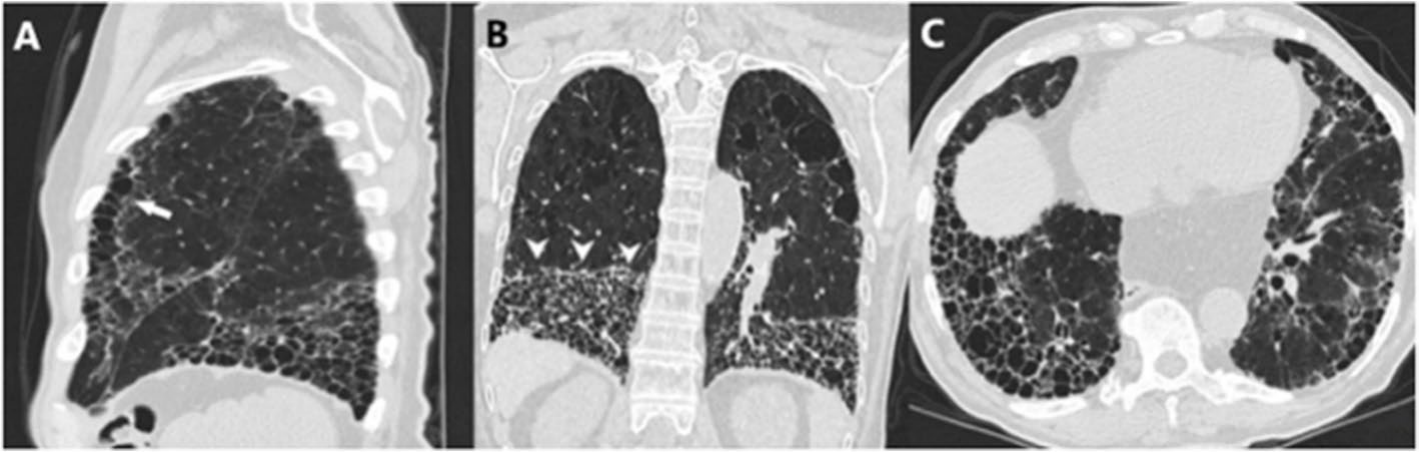
Radiological ancillary findings in ARDs-related UIP pattern **A**) Marked honeycombing/reticulation both primarily  in the lower lobe. However, a distinct “anterior upper lobe” sign is visible (white arrow) in a patient with RA-UIP. **B**)  “Straight edge” sign with in a patient with polymyositis related UIP pattern. **C**) Axial image from the same patient as A,  both the axial and sagittal images have almost exclusively honeycombing representing the “exuberant honeycombing” sign

*The “anterior upper lobe” sign* is defined as concomitant fibrosis with honeycombing in the anterior part of the upper lobe combined with a UIP pattern consisting of reticulation and honeycombing in a predominantly basal distribution (Fig. 2A). It is found in 25.4–34.9% of patients with ARD-UIP compared to 12.8–17.2% of patients with IPF-UIP.

*The “straight-edge” sign* is a markedly sparing of the upper and midzones from fibrosis with a sharp horizontal demarcation between the fibrotic and normal lung parenchyma (Fig. 2B). Furthermore, the fibrosis does not extend cranially along the lateral chest wall. Patients with ARD-UIP exhibit the sign in 25.4–36.0% of the cases, whereas only 6.0–8.3% of patients with IPF-UIP [31]. The “straight-edge” sign is associated with an increased survival, mainly in patients where the sign is found in combination with IPF-UIP. It has been suggested that this could represent a certain phenotype of IPF.

*The*” *exuberant honeycombing” sign* is characterized by more than 70% of the fibrotic lung and it is made up by honeycomb cysts (Fig. 2C). This sign can be described in 22.2% of patients with ARD-UIP compared to 6.0% with IPF-UIP.

Furthermore, the presence of pulmonary nodules [32], unilateral pleural effusion [33], pleural thickening, minimal pericardial effusion combined with pericardial thickening [34] and bone involvement in the form of erosions, soft tissue swelling, subchondral cyst formation and demineralization combined with a radiological UIP pattern should raise the suspicion of a connective tissue disease (CTD)-UIP.

Recently, advances in quantitative imaging have suggested that a parameter measuring the vessel-related structure volume (VRS), where the vessels are anatomically segmented by an algorithm and the full volume is extracted, is a prognostic factor of patient outcome. Furthermore, a study by Chung et al. found that a higher VRS was associated with IPF compared to ARD-ILDs though not differentiating on the radiological pattern of the disease [35].

## Pathogenesis of the UIP pattern in IPF and autoimmune rheumatic diseases

As mentioned above, the UIP pattern is the most frequently observed in RA-ILD [36, 37]. Moreover, RA-UIP and IPF have a poor prognosis, with a similar median survival rate: in a CT comparison between IPF and rheumatoid lung disease, patients with IPF and patients with RA-UIP both had a 4-year survival of approximately 35% [16].

Both clinical similarities and common environmental risk factors support the hypothesis of a shared genetic background in RA-ILD and IPF. In agreement with this hypothesis, an excess of rare variants in genes linked to familial pulmonary fibrosis has been detected in RA-ILD as compared with controls [38]. An excess of pathogenic variants were also observed in telomere maintenance genes (TERT, PARN, RETL1) and in SFTPC, involved in surfactant homeostasis [38]. An increased magnitude of the association was observed in patients with a UIP pattern [36].

Fhe functional MUC5B rs35705950 promoter variant, which is the major risk factor for IPF [14], was recently identified as a risk factor for RA-UIP, whereas it was not associated with RA without ILD. This observation provides definitive evidence for a common genetic architecture in RA-UIP and IPF [14, 15].

A recent study reported that RNA sequencing of lung biopsies from patients with RA-ILD and IPF revealed shared and distinct disease-causing intracellular pathways [39]. In fact, analysis of transcriptomic data identified a JAK2 related JAK/STAT signaling pathway gene signature that distinguishes RA-UIP from idiopathic UIP. This was further confirmed by immunohistostaining, which identified JAK2 phosphorylation with two distinct forms of activation: a cytoplasmic form of JAK2 activation in most IPF cases and a nuclear form of p-JAK2 in RA-UIP and a minority of IPF cases. Further immunohistostaining identified STAT5A&B as the downstream transcriptional activator for JAK2-mediated canonical signal transduction and phosphorylation of Tyr41 on histone H3 (H3Y41ph) as the downstream epigenetic regulation site for JAK2-mediated noncanonical signal transduction. Gene Set Enrichment Analysis (GSEA) of the RNA-Seq data further supported this shared pathogenic mechanism for the two diseases with the enrichment of STAT5 A&B target gene sets as well as the JAK2 regulated H3Y41ph target gene set.

This regulatory role of JAK2 in the pathogenesis of pulmonary fibrosis was further demonstrated by the attenuation of bleomycin-induced murine pulmonary fibrosis using a JAK2-selective pharmacological inhibitor CEP33779 [39].

## Epidemiology, risk factors and natural history

Similar to idiopathic UIP, the most consistently reported risk factors for development of a UIP pattern in patients with ARD-ILD include older age, male gender and smoking [40]. Specific risk factors for RA-ILD include positive anti-cyclic citrullinated peptide antibodies or IgM rheumatoid factor, MUC5b polymorphisms, and, in some studies, RA disease activity [41, 42]. Smoking is the only preventable risk factor.

Although reported estimates of ARD-ILD may vary depending on the methodology utilized for diagnosis, in a Danish study, using prospectively collected data from population-based databases, clinically significant CTD-ILD is identified in about 5% of patients with CTD [43] whereas the prevalence increases to 19% when patients are screened by HRCT [44]. However, there are no specific data regarding the incidence and prevalence of a UIP pattern in CTD-ILDs in general.

RA is the most common ARD, with a prevalence of 1–2% in the general population; it commonly occurs in women, with a female to male ratio equal to 3:1. However, in RA-ILD, the female to male ratio is closer to 1:1. Pulmonary manifestations including ILD, chronic obstructive pulmonary disease and bronchiectasis occur in up to 80% [45].

Recently, Juge and colleagues developed a scoring system that allows stratification of patients at high risk for RA-ILD before the onset of their pulmonary symptoms (i.e., subclinical RA-ILD) with the aim to help clinicians to identify patients who would most benefit from screening. They proposed and validated a risk score for subclinical RA-ILD that included 4 variables: sex, age at RA onset, RA disease activity using DAS28-ESR (disease activity score on 28 joints, calculated with erythrosedimentation rate), and the MUC5B rs35705950 genetic variant. Although the risk score without MUC5B rs35705950 was found to be appropriate to discriminate patients with subclinical RA-ILD, the model with MUC5B rs35705950 had better performance, suggesting a slight contribution of the genetic variant to the overall risk of subclinical RA-ILD [42].

Moreover, in a recent systematic review, the UIP pattern on HRCT was one of the main risk factors associated with mortality among RA-ILD patients, together with older age, male sex, smoking history, lower diffusing capacity for carbon monoxide percentage (DLCO%) predicted, lower forced vital capacity percentage (FVC%) predicted, emphysema, and acute exacerbations (AE) of RA-ILD [46]. Similarly, the UIP pattern represents a risk factor for the occurrence of AE-ILD, further characterizing UIP as a major risk factor for progression and/or mortality in these patients.

The prevalence of ILD in SSc patients varies widely between 34 and 60% [47]. ILD is more common in diffuse compared to limited SSc with a reported prevalence ranging from 40 to 71% and 21–-53%, respectively [47]. In addition, ILD occurs more frequently in patients with positive anti-topoisomerase I (anti-Scl-70) antibody, but may also be present in patients with other SSc specific antibodies [47]. In SSc-ILD, NSIP is the most prevalent histological pattern and UIP is a relatively less common pulmonary manifestation of SSc [22]. SSc-ILD has a variable clinical course. Most patients will experience a slow decline in lung function, but some progress rapidly after disease onset [48]. However, there is no evidence that the pattern of fibrosis on HRCT or histology (e.g., NSIP versus UIP) has a significant impact on disease progression or mortality in patients with SSc [49].

The UIP pattern is less prevalent in polymyositis and dermatomyositis [20] and it is probably rarer than NSIP in *systemic lupus* erythematosus (SLE), based on anecdotal clinical experience [22]. Historical data on pSS [50] report a higher prevalence of NSIP, but recent data on non-sicca onset of pSS suggest a higher prevalence of UIP pattern [27]. Furthermore, it is not clear, whether the histological distinction between UIP and NSIP has prognostic importance in these disorders [51].

## Diagnosis and treatment decisions are based on a multidisciplinary team approach

Patients with a HRCT UIP pattern are most often referred for pulmonologist evaluation. Here, two scenarios are frequent: first, patients can be referred from other centers or general physicians due to respiratory symptoms and HRCT findings without any known underlying disease association. Here, the pulmonologist has to thoroughly evaluate the patient for signs or symptoms suggestive of any ARD [52]. If suspicion of an ARD, the patient should be referred for rheumatologist evaluation. Second, patients are referred from the rheumatologist with a known ARD and new or progressive respiratory symptoms on the suspicion of an ILD. Here, the pulmonologist should evaluate the HRCT and patient history to make a diagnosis of ARD-ILD.

Making the final diagnosis is best obtained by a multidisciplinary team approach (MDT) involving pulmonologist, rheumatologist and radiologist. Surgical lung biopsies in patients suspect of an underlying ARD is not needed if a UIP pattern and is related to a higher mortality risk and execution should therefore be carefully discussed at the MDT [53]. Lung tissue by either transbronchial cryo-biopsies or a surgical lung biopsy is only rarely needed in a diagnostic perspective as, at the moment, histologic patterns do not provide further guidance on prognosis or treatment decisions. However, this may change in the future if and when a more personalized approach, like seen in oncology, will gain attraction. Obviously, a pathologist should participate in MDT, if a lung biopsy has been taken.

At the MDT, the diagnosis of ARD with a UIP pattern should be confirmed, and the disease severity based on the integration of symptoms, pulmonary function impairment and morphological extent of disease on HRCT. Potential extrapulmonary organ involvement is discussed including the presence of pulmonary hypertension to form basis for prognostication and treatment decisions. In a patient with ARD-UIP, management of lung disease should be discussed according to the other possible systemic manifestation of the ARD.

## Evaluation of disease severity and progression

In ARD-ILD, an evaluation of severity, progression and response to treatment is based on the combination of changes in symptoms, pulmonary function trends, and, in selected patients, serial CT evaluation [22, 54]. The integrated evaluation of these different items should be performed during a multidisciplinary discussion [22]. Especially for rheumatic diseases, PFTs are considered to be superior to symptoms in evaluating ILD involvement since extrapulmonary manifestations of the disease may be responsible for major exercise intolerance due to the increased work of locomotion. Similarly, major musculoskeletal limitations may mask ILD-related exertional breathlessness [54].

The most accurate tool for estimating ARD-ILD progression is focused on serial PFTs. Since FVC is highly reproducible, in the absence of major extrapulmonary restriction due to pleural disease, muscle weakness, or cardiac disease (particularly, congestive heart failure), changes in FVC are specific to ILD [55].

In patients with SSc, Goh and colleagues introduced a simple staging system whereby an HRCT disease extent of > 20% defined extensive rather than limited ILD and was strongly associated with mortality. Where the extent of disease on HRCT was indeterminate, the use of an FVC threshold of 70% allowed separation into limited and extensive disease [56].

An impairment in DLCO that is disproportionate to lung volumes may suggests the coexistence of underlying pulmonary hypertension [22]. However, a preservation of lung volumes together with a severe reduction in DLCO (ratio of FVC% predicted to DLCO% predicted > 1.6) can also indicate the coexistence of ILD and emphysema in ever-smoking patients with ARD [57]. Therefore, in the individual patient, this pulmonary function profile requires the interpretation of HRCT and echocardiographic data during a multidisciplinary discussion.

Several studies have shown that 6-minute walk distance (6MWD) and/or decline in 6MWD are strong independent predictors of mortality in patients with IPF [58–60] and other ILDs [61, 62]. The occurrence of desaturation (SpO2 ≤ 88%) during or at the end of a 6MWD and change in SpO2 during a 6MWD have been found to be significant predictors of mortality [63]. Both baseline 6MWD < 250 m and a decline of 50 m from baseline at 24 weeks 6MWD were associated with a significant increased mortality risk [64]. However, exercise limitation in ARD-ILD can be considered multifactorial, with contributions including impairment of gas exchange and pulmonary hypertension, ventilatory dysfunction and musculoskeletal disease [65]. Particularly, pulmonary hypertension is considered a frequent complication in SSc and mixed connective tissue disease, whereas it is much rarer in RA, systemic lupus erythematosus and myositis, and is when present, a marker of poor prognosis [66].

Patient-reported outcomes are important for measuring disease progression in IPF whereas evidence is sparse in ARD-ILDs. St George’s Respiratory Questionnaire, a measure of respiratory-related health status often used in IPF, in ARD-ILDs, and specifically SSc-ILD, may be affected by comorbidities, including musculoskeletal problems.

Finally, increasing evidence suggests a role of lung ultrasound as a prognostic marker for the appearance or worsening of ARD-ILD. In two studies the number of baseline B lines has a good accuracy to predict the worsening of both DLCO and HRCT during follow-up [67].

Some of these tools have been evaluated only in specific ARD-ILD, therefore their generalizability is debatable.

## Pharmacological treatment

Historically, immunosuppressants have been considered the cornerstone of therapy for patients with ILDs other than IPF, irrespectively of the radiologic or histologic pattern. The degree of evidence for the available immunomodulatory drugs in the specific inflammatory rheumatic diseases such as corticosteroids, mycophenolate mofetil (MMF), azathioprine, methotrexate, cyclophosphamide and rituximab are sparse, except in SSc even though these therapies are often employed [68]. However, a recent retrospective study, in patients with RA who started treatment for ILD with mycophenolate, azathioprine, or rituximab, observed that immunosuppression was associated with an improved trajectory in FVC and DLCO compared with the pretreatment pulmonary function trajectory, regardless of the kind of drug. Interestingly, patients with a UIP pattern of ILD in immunosuppressive treatment did not show a worse pulmonary function trajectory as compared with patients with a non-UIP ILD [21].

The recent demonstration of efficacy of the antifibrotic drug nintedanib in progressive fibrosing ILDs other than IPF has introduced a new therapeutic approach to ARD-ILD supplementary to those already used [69].

Recently, official clinical practice guidelines for the management of IPF and PPF in adults have been developed [70]. Therefore, the ILD subset, mainly the radiological/histological pattern and the degree of fibrotic component, other than the clinical behaviour of ILD, is becoming increasingly important when determining the therapeutic strategy also for patients with ARD-ILD [71].

The two antifibrotic drugs nintedanib and pirfenidone were proposed for the treatment of these patients with different degrees of evidence [72].

Nintedanib acts as a triple tyrosine kinase inhibitor, simultaneously inhibiting signaling pathways activated by platelet-derived growth factor (PDGF), fibroblast growth factor (FGF), as well as vascular endothelial growth factor (VEGF) [73]. In contrast, the exact mechanism of action of pirfenidone is not fully understood. It is suggested that the antioxidant effects of pirfenidone contribute to its anti-inflammatory effects, leading to antifibrotic effects [74]. Pirfenidone attenuates the production of transforming growth factor-β1 (TGF-β1), a key profibrotic and pro-inflammatory cytokine implicated in idiopathic pulmonary fibrosis (IPF). Some evidence also suggests that pirfenidone downregulates pro-inflammatory cytokines, including TNF-α, interleukin-1 (IL-1), IL-6, interferon-gamma (IFN-γ) and platelet-derived growth factor (PDGF) [75].

Currently, four randomized clinical trials (RCTs), one for nintedanib and three for pirfenidone, have evaluated the efficacy of antifibrotic drugs in patients with fibrosing ILD, including patients with UIP pattern. Unfortunately, the designs, the number and the diagnoses of enrolled patients were different among the 4 RCTs and their results are not comparable and some are inconclusive.

The recent ATS/ERS/JRC/ALAT guidelines conditionally recommended nintedanib for the treatment of PPF in patients affected by ILDs other than IPF who have failed standard management. Notably, standard management was not further defined. The INBUILD study demonstrated the efficacy of nintedanib in patients affected by PPF other than IPF, with highest efficacy in patients with a UIP pattern; in fact, in these latter, the difference in the annual decline in FVC between nintedanib and placebo arms was 128 ml/year, while it was 75.3 ml/year in patients with a radiological non-UIP pattern [76]. Concurrently, nintedanib decreased the risk of acute exacerbation by 2.3 times among patients with radiological UIP pattern, whereas no difference in acute exacerbation risk was observed among patients without a UIP pattern [76]. The number of patients with ARDs and a radiologic UIP pattern was too small to allow definitive conclusions about the efficacy of nintedanib in these patients.

The ATS/ERS/JRC/ALAT guidelines did not make any treatment recommendations for or against pirfenidone but recommended further research into the efficacy, effectiveness, and safety in non-IPF ILD patients manifesting PPF based on three different RCTs evaluating the efficacy of pirfenidone in unclassifiable ILD, PPF other than IPF (RELIEF study) and RA fibrosing ILD (TRAIL1 study) [77–79].

In a trial focusing on fibrotic unclassifiable ILD, pirfenidone did not meet the primary endpoint, the decrease in mean change by FVC measured by daily home spirometry over 24 weeks compared to placebo; however, the results of key secondary endpoints suggested possible benefit from the drug [77]. The RELIEF and TRAIL1 studies were both prematurely interrupted because of slow recruitment and futility.

**Fig. 3:**
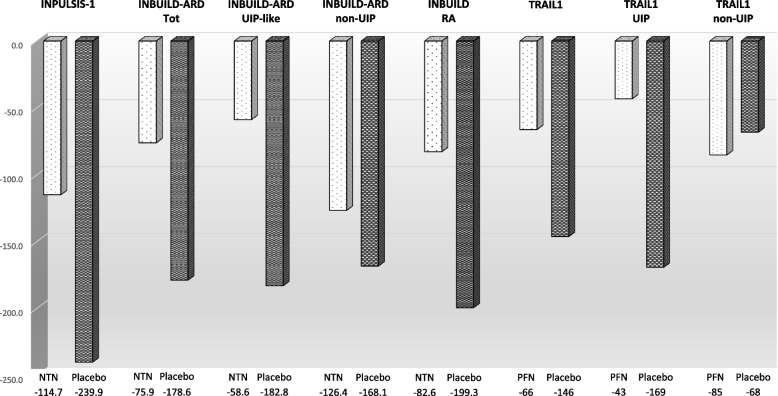
Decline of forced vital capacity (FVC) in treated and placebo groups from INPULSIS [83], RA and ARDs subgroup from INBUILD [84], and TRAIL1 [67] trials, according to UIP/non UIP pattern. Data were reported as reduction in ml/52 weeks. In INBUILD study, 86.5% of RA patients had a UIP-like fibrotic pattern on HRCT (sub-analysis for UIP/non UIP pattern was not performed)

In the RELIEF trial, imputations were conducted for missing data with the primary analysis favouring the pirfenidone arm [78]; the TRAIL1 study failed in reaching the primary outcome (a composite endpoint of a decline from baseline in FVC of 10% or more or death), but pirfenidone was associated with a clinically significant slower rate of FVC decline in patients with RA-ILD compared to placebo [79]. In particular, the subgroup analysis suggested that pirfenidone could be more effective in patients with RA-UIP (estimated mean decline from baseline − 126 mL vs – 17 mL for patients with UIP and non-UIP, respectively) [79]. Contrary to the INBUILD trial, TRAIL1 was not enhanced by the inclusion of patients with a progressive behaviour of ILD, indirectly suggesting that RA-UIP should always be considered as a progressive disease (Fig. 3).

Various emerging treatments are being investigated in patients with progressive pulmonary fibrosis, including also patients with ARD-ILD [80].

ARDs are systemic diseases with possible pulmonary and extra-pulmonary manifestations; therefore, treatment of lung involvement needs to take into consideration other potential extrapulmonary manifestations. For this reason, combination therapy, including steroids, immunosuppressive drugs, conventional synthetic-, biologic- or targeted synthetic-disease modifying anti-rheumatic drugs (DMARDs) are often used for the treatment of these patients. Some case reports already described combination therapy with DMARDs and antifibrotic drugs [81, 82] and some patients enrolled in the INBUILD, TRAIL1, and RELIEF trials were also treated with immunosuppressants or DMARDs, indirectly confirming, in a small number of patients, the possible safety of combination therapy [76]. On the other hand, SENSCIS [83] and LOTUSS [84] studies demonstrated the safety of a combination therapy between mycophenolate mofetil and nintedanib or pirfenidone.

As reported above, some authors had previously described a possible effect of DMARDs on RA-ILD [85, 86]. Particularly, abatacept has been associated to a lung function improvement in a significant percentage of RA patients complicated by ILD regardless of ILD pattern [87]. Also, in smaller studies, other biologic DMARDs showed similar effects [81, 88–90].

In patients with systemic autoimmune diseases, including RA, different degrees of inflammation and fibrosis could contribute to the lung damage, even in patients with a UIP pattern [37, 41]. Therefore, immunosuppressants might reduce lung inflammation with a nonspecific modality of action, alone or with an additive or synergistic effect with prednisone and DMARDs.

The lack of data regarding lung function trajectories in RA-ILD does not allow to discriminate the effect of DMARDS, such as abatacept, rituximab, tocilizumab and Janus kinases inhibitors and immunosuppressants, in contrast to the physiological, “untreated” natural history of RA-ILD. This hypothesis is also suggested by the very similar effect of DMARDs among the different studies evaluating these drugs in RA-ILD [91]. Supporting this hypothesis, Selman and colleagues recently suggested that the UIP pattern in RA could share pathogenic and clinical-radiological features with IPF, representing a unique entity with similar progression over time and therapy response [37]. This hypothesis is also supported by the observation that UIP in RA-ILD patients enrolled in INBUILD and TRAIL1 studies showed a FVC decline similar to IPF [76, 81]. However, we cannot exclude that, differently from IPF, UIP in RA-ILD might benefit from a combination therapy including antifibrotic drugs, DMARDs and/or immunosuppressants.

The variability in clinical phenotype of ARDs and the absence of guidelines on rheumatological diseases reflect the heterogeneous, and sometimes conflicting, therapeutic algorithms proposed by different authors [92]. Therefore, a multidisciplinary approach, including at least rheumatologist, pulmonologist, and radiologist is desirable for the management of ARD-ILD patients and should balance a high level of need for treatment with the risk of adverse events from multi-morbidities and -therapies until more evidence is present [92–95].

## Data Availability

Not applicable.

## Abbreviations

AE: Acute exacerbations
ARD: Autoimmune rheumatic diseases
CTD: Connective tissue disease
DLCO: Diffusing capacity for carbon monoxide
FVC: Forced vital capacity
HRCT: High-resolution computed tomography
IIP: Idiopathic interstitial pneumonias
IPF: Idiopathic pulmonary fibrosis
ILD: Interstitial lung diseases
DMARDs: Modifying anti-rheumatic drugs
MMF: Mycophenolate mofetil
MDT: Multidisciplinary team approach
NSIP: Nonspecific interstitial pneumonia
OP: Organizing pneumonia
pSS: Primary Sjögren’s syndrome
PFTs: Pulmonary function tests
PPF: Progressive pulmonary fibrosis
RA: Rheumatoid arthritis
SLE: *Systemic lupus* erythematosus
SSc: Systemic sclerosis
UIP: Usual interstitial pneumonia
VRS: Vessel-related structure volume
6MWD: 6-minute walk distance
